# A Codesign Framework for the Development of Next Generation Wearable Computing Systems

**DOI:** 10.3390/s25216624

**Published:** 2025-10-28

**Authors:** Francesco Porreca, Fabio Frustaci, Raffaele Gravina

**Affiliations:** Department of Computer, Electronics, Modeling and Systems Engineering, University of Calabria, 87036 Rende, Italy; f.frustaci@dimes.unical.it

**Keywords:** FPGA, HW/SW codesign framework, wearable computing system, IoT, green electronics

## Abstract

Wearable devices can be developed using hardware platforms such as Application Specific Integrated Circuits (ASICs), Graphics Processing Units (GPUs), Digital Signal Processors (DSPs), Micro controller Units (MCUs), or Field Programmable Gate Arrays (FPGAs), each with distinct advantages and limitations. ASICs offer high efficiency but lack flexibility. GPUs excel in parallel processing but consume significant power. DSPs are optimized for signal processing but are limited in versatility. CPUs provide low power consumption but lack computational power. FPGAs are highly flexible, enabling powerful parallel processing at lower energy costs than GPUs but with higher resource demands than ASICs. The combined use of FPGAs and CPUs balances power efficiency and computational capability, making it ideal for wearable systems requiring complex algorithms in far-edge computing, where data processing occurs onboard the device. This approach promotes green electronics, extending battery life and reducing user inconvenience. The primary goal of this work was to develop a versatile framework, similar to existing software development frameworks, but specifically tailored for mixed FPGA/MCU platforms. The framework was validated through a real-world use case, demonstrating significant improvements in execution speed and power consumption. These results confirm its effectiveness in developing green and smart wearable systems.

## 1. Introduction

Wearable Computing Systems (WCSs) are sensor-based systems with computational capabilities that collect data from the user wearing them. Formally, such devices are composed of three main parts [1]: (a) sensors, which can be worn under/over/embedded in clothing and are responsible for collecting physiological and behavioral data from the user interacting in the environment; (b) a computational resource that in addition to acquiring data from the sensors processes them by providing (depending on computing power) smart functionality; (c) a wireless communication module in order to connect these devices with the rest of the IT ecosystem. Thanks to their versatility, devices like smartwatches, fitness trackers, and smart clothing have become widely used in various application fields, including healthcare, entertainment, sports, and Ambient Assisted Living (AAL). These systems not only enhance user experience but also provide valuable insights into health and performance metrics. Furthermore, the integration of advanced algorithms and machine learning techniques can enable predictive analytics and personalized recommendations, making WCS an essential tool in modern technology.

Modern wearable applications often rely on microcontroller-based platforms due to their low cost and flexibility. While software-only approaches offer ease of development, they struggle to meet the growing computational demands of applications involving machine learning and real-time processing. High-performance alternatives like GPUs are typically unsuitable because of their high energy requirements, conflicting with the power constraints of wearable devices. Offloading computation to the cloud can mitigate local limitations but introduces latency and privacy concerns. These challenges highlight the need for more efficient, edge-capable solutions beyond the traditional software-only paradigm.

To enable standalone wearable devices without relying on cloud or smartphones, this work presents COCOWEARS: a framework for designing next-generation systems based on hybrid FPGA/MCU platforms. By leveraging hardware/software co-design, the framework simplifies development while optimizing energy use, speed, and processing power. Table 1 provides a comparison with traditional techniques used for the development of wearable systems, highlighting the advantages of the proposed approach. Introducing the hardware/software co-design paradigm in the context of WCS system development involves the adoption of heterogeneous System-on-Chip (SoC) platforms integrating, on the same die, a general-purpose processor and a reconfigurable logic fabric [2]. The former enables flexible system control by leveraging the adaptability typical of software-based procedures. It can also host routines that are not critical in terms of execution speed, such as the management of data acquisition from multiple sensors. The latter, instead, provides the capability to implement computational cores directly in hardware, which, if executed in software, might not satisfy the real-time execution requirements typically imposed by such applications, nor comply with the available power budget. For this reason, the primary advantage targeted by the proposed framework is the acceleration of data processing procedures to guarantee compliance with real-time constraints. Such a requirement is not always achievable when using existing frameworks, which are typically designed as a collection of software routines executed on hardware platforms characterized by limited computational resources, such as microcontrollers, mainly due to constraints on power dissipation and/or physical footprint [3,4,5,6]. Mapping processing procedures onto hardware allows one to overcome the inherently sequential nature of execution typical of such computational platforms and to fully exploit the principle of parallel computing, which can be effectively implemented in hardware. This is achieved through the instantiation of multiple hardware accelerators within the programmable logic section, which—by not sharing hardware resources among each other (except, depending on the application, communication buses toward memory and/or peripheral devices)—are capable of performing their respective tasks concurrently. In addition, designing a hardware circuit specialized for performing a specific operation also enables energy savings compared to executing the same operation in software on a non-specialized, general-purpose architecture [7]. At the same time, hardware accelerators provide higher computational power and facilitate the implementation of the edge computing paradigm, in which data are processed directly at the acquisition site (i.e., where they are collected), without the need to be transferred to network nodes with higher computational capacity [8]. This approach not only avoids additional transmission latencies and potential application bottlenecks but also enhances the security of the processed data.

The applicability and benefits of COCOWEARS were evaluated using real testbeds and simulations that will be described throughout this paper. As a result, it will be shown that the proposed framework enables a reduction in the execution time of the k-nearest neighbor (k-NN) algorithm–used for user activity recognition applications–by up to approximately 96× compared to existing frameworks that rely solely on modular software-based procedures. Similarly, it will be demonstrated that the proposed approach also achieves a reduction in the dynamic energy dissipated by the computing platform by up to approximately 50×.

### Related Work

A significant portion of research endeavors has focused on the use of System on Chip (SoC) in wearable devices due to their ability to optimize performance, such as battery consumption and processing efficiency. SoCs, which integrate both a general-purpose CPU (Central Processing Unit) and FPGA (Field-Programmable Gate Arrays) provide flexible and high-performance platform for advancing the next generation of wearable devices across various fields, including industrial application, medical technology, sports, Human Assisted Living (HAL), and military operations [9]. Development of these devices is very complex and requires a wide range of knowledge in a variety of areas, this very often results in lack of code reusability and poor system modularity.

Some of the discussed work demonstrates how the use of FPGAs successfully meets the computational necessities often required by wearable devices. In the medical field, for example, ultrasound image processing demands significant computational power for real-time data elaboration. While high-performance CPUs can handle these tasks, they require high power dissipation, making them unsuitable for wearable systems and long-term monitoring. The work of [10] shows that implementing an ultra fast beamformer system on an FPGA can effectively meet these computational demands while keeping the device’s power dissipation in check. This makes FPGAs a viable solution for developing efficient and sustainable wearable medical devices.

Often, when considerable computational power is required, such as for the execution of complex machine learning algorithms, data are sent over the Internet network to the Cloud. These, once processed, then return to the device. The need for an Internet connection, bandwidth consumption, privacy concerns and speed of system response have led toward the development of edge-type systems that process data close to where they are produced. To meet the computational demands of these algorithms, FPGA devices are extremely suitable, as demonstrated in this other study [11] again applied to the medical field, where an FPGA-based device is developed for acquires data and processes it via the CNN algorithm. From the results they obtained, it was shown not only that FPGA succeeds in meeting the requirements of the application, but is also 12× faster than the software-developed system.

The presented works all develop systems requiring high computational power, which could not be achieved on a standard microcontroller without relying on cloud-based processing. To enable far edge devices, it is essential to adopt more advanced hardware platforms. However, to the best of our knowledge, no dedicated framework currently exists for developing wearable systems through hardware-software co-design techniques. Developing such a framework is crucial, as it simplifies application development, reduces errors, and allows developers to focus on the creation itself rather than the intricacies of implementation.

In the context of Body Sensor Networks (BSNs), several frameworks have been proposed to address challenges related to data processing, communication, and system integration. One notable example of these efforts is CodeBlue [6], a framework designed for wireless medical monitoring, enabling real-time patient tracking and emergency response in healthcare environments. It focuses on low-power communication and sensor data aggregation. Another example is represented by Titan (TIny TAsk Network) [5], a signal-processing-oriented framework supporting efficient data fusion and adaptive processing for various health monitoring scenarios. A more recent framework, WatchOverGPT [12], is designed for real-time crime detection and response using wearable cameras and large language models (LLMs). It operates across three key levels: *Data Acquisition*—Wearable sensors and cameras continuously capture images and videos from the environment. *AI Processing*—A powerful LLM analyzes visual and contextual data, identifying suspicious behaviors or threats. *Autonomous Decision-Making*—The system alerts authorities or provides safety recommendations in real-time. Similarly, BlockTheFall [13] is a framework developed for real-time fall detection in elderly care. It follows the same three-tier approach: *Data Acquisition*—Wearable sensors continuously monitor movement and posture to detect balance irregularities. *AI Processing*—Machine learning algorithms analyze sensor data to distinguish between actual falls and normal movements, enhancing detection accuracy. *Secure Data Management*—Blockchain technology ensures data integrity and security, reliably recording fall events for verification and emergency response. Beyond detection, BlockTheFall provides guidance for developers, helping them identify the most relevant aspects of a fall detection application, such as sensor selection, alert mechanisms, and data processing techniques, ensuring an optimized and effective implementation.

SPINE [3] and its task-oriented redesign, SPINE2 [4], are domain-specific software frameworks for the design of Wireless Body Sensor Network Applications. SPINE promote code reusability and modularity. It provides APIs and libraries which simplify the development process of signal processing algorithms for sensor data, distributed under an Open Source license, it aims to establishing a broad community of users and developers who contribute to the scientific evolution of the framework.

However, existing frameworks focus exclusively on the software layer and are typically built for specific MCU-based platforms, limiting flexibility.

Previous frameworks such as SPINE, in fact, propose a modular design based on the (re-)use of basic components necessary for the development of wearable embedded systems, yet relying solely on software routines. The proposed COCOWEARS framework instead, as a key distinguishing feature, aims at the design of a system based on a “meet-in-the-middle” approach based on hardware–software co-design space exploration. This choice enhances both performance and energy efficiency.

## 2. Materials and Methods

This section introduces the COCOWEARS Framework, a novel approach for the efficient and accelerated development of next-generation wearable devices.

The importance of a dedicated framework for wearable system development lies in its ability to optimize design efficiency across multiple crucial aspects:

- *Computational Power*: Wearable systems need to process data locally to guarantee real-time performance and minimize latency. Relying on cloud services can introduce delays, connectivity issues, and security risks. A well-structured framework allows for optimized hardware and software, maximizing local processing power for a seamless user experience;
- *Speed*: Real-time processing is crucial for wearable applications, such as health monitoring or gesture-based control. An effective framework helps balance resources between hardware and software, ensuring that computations are executed with maximum efficiency while keeping power consumption low;
- *Energy Efficiency*: Beyond battery life, the overall energy consumption of electronic devices is a growing concern. Thinking in terms of Green Electronics, it means reducing environmental impact, enhancing sustainability, and optimizing power efficiency on a global scale. A well-designed framework ensures that wearable devices are high-performing, eco-friendly, and energy-efficient, without compromising functionality or reliability.

The foundation on which this framework is designed relies on the Platform Based Design (PDB) [14] and the SPINE Framework [3]. Platform-Based Design is a structured methodology that defines design as “meet-in-the-middle” refinement process, where system specifications guide the selection of the most suitable platform. This approach ensures an optimal balance between flexibility and efficiency. The SPINE Framework is designed to enhance the modularity and reusability of software components and hardware accelerators, facilitating their integration into complex systems.

### 2.1. The Proposed Framework

In COCOWEARS, PBD inspired the definition of constraints, adopting a top-down approach to guide platform selection based on factors such as speed, power dissipation, and hardware availability. Meanwhile, SPINE influenced the architecture of software components and hardware accelerators, ensuring their reusability and adaptability across different implementations. Unlike frameworks purely software-oriented like SPINE, COCOWEARS must deal with the inherent complexity of heterogeneous systems—i.e., supporting both software routines and hardware accelerators, each with different execution paradigms, resource constraints, latency profiles, and communication fabrics. Because of this, a major part of our effort has been to model and conceptualize an architecture and interface abstraction capable of working seamlessly with both paradigms. The COCOWEARS Framework is designed to optimize the development of wearable systems by systematically selecting the most suitable platform. The platform selection process is based on high-level constraints defined by the developer, who specifies requirements such as computation speed, delay, occupied area (memory footprint for software components and chip area for FPGA implementations), power consumption, and the tasks to be performed (e.g., machine learning classification, signal filtering, or data compression). The developer does not need prior knowledge of the optimal execution platform, as COCOWEARS aims to autonomously determine the best deployment based on application constraints and operations. While full automation is the long-term goal, the current focus is on defining the methodology and architecture. Central to this approach are high-level interfaces that abstract communication with both hardware accelerators and software components. These components must be pre-built, characterized, and made available in a repository to enable future automated platform selection and seamless task migration with minimal developer intervention.

#### 2.1.1. Repository-Driven Platform Selection

At its core, COCOWEARS operates based on a structured repository of pre-implemented hardware and software components to make informed platform selection decisions. This repository is conceptually divided into two sets:

- *Hardware Components Set*—Contains pre-implemented modules designed for FPGA-based acceleration, such as signal filtering blocks, matrix operations, machine learning classifiers (e.g., KNN), or control units.
- *Software Components Set*—Includes implementations that run on CPUs/MCUs, handling tasks such as digital filtering (e.g., IIR filters), basic classification algorithms, and general-purpose processing.

Ideally, for each operation, the repository should contain both hardware and software implementations, ensuring a one-to-one correspondence between the two sets. This allows COCOWEARS to analyze existing components, compare their performance metrics (e.g., latency, resource usage, power consumption), and determine whether a task should be implemented in hardware or software.

However, if only one implementation instance (hardware or software) exists, the framework will work with the available option. If a hardware implementation is missing, the framework suggests either using the software alternative or guiding the developer in implementing a hardware version, following structured design guidelines for reusability. If a software implementation is absent, the developer may need to rely on an FPGA-based solution or create a software alternative within the platform’s constraints. By structured design guidelines, we mean that every module—whether hardware or software—must interact with the rest of the system through a common, well-defined interface. In practice, this means that a hardware accelerator and its software counterpart expose identical input/output contracts, data formats, handshaking semantics, and control signals. Because both variants “speak the same language” at the architectural boundary, they can be swapped or replaced without altering the overarching system flow, thus preserving consistency and enabling seamless fallback or hybrid execution.

After this evaluation process, COCOWEARS suggests how to interconnect the selected components, ensuring an optimized workflow where software does not introduce bottlenecks that limit hardware efficiency. This structured approach guarantees seamless integration, high performance, and energy-efficient execution of wearable systems.

At the end of the selection process, COCOWEARS provides the developer with recommendations on the most suitable approach for implementing the wearable system, choosing from three alternatives:

- *Software-Only Solution*: The CPU is responsible for both data management and processing, as no hardware accelerators are present. This solution prioritizes flexibility and ease of development, making it ideal for scenarios where high computational power is not required.
- *Hardware-Only Solution*: The CPU serves solely as a coordinator, overseeing data acquisition, task distribution, and system control, while all computational workloads are executed on the FPGA hardware accelerators. This approach optimizes speed and latency, making it suitable for applications requiring significant processing power. However, due to the complexity of FPGA development, this solution may not always be accessible to all developers.
- *Hybrid Approach*: The CPU acts as a coordinator and also performs processing, working alongside FPGA-based accelerators to balance computational efficiency, energy consumption, and scalability. By leveraging both architectures, this model ensures optimized execution performance while maintaining ease of development, making it a strong compromise between the software-only and hardware-only solutions.

A fundamental principle of the COCOWEARS Framework—beyond guiding the developer in platform selection—is its emphasis on code and component reusability. By leveraging a structured repository of pre-implemented hardware and software components, developers can significantly simplify the development process, making it more adaptable to new requirements while maintaining efficiency. This approach reduces time to market, as developers can reuse validated and characterized components rather than designing them from scratch. Additionally, it minimizes potential errors, ensuring that previously tested modules contribute to greater system reliability. Furthermore, the flexibility and scalability of the framework allow developers to build more complex and efficient systems, while maintaining the ability to extend functionality as needed. Whether integrating new components or refining existing ones, COCOWEARS ensures a structured, reusable, and optimized development cycle, making wearable system design more accessible and effective.

All materials developed so far, including the hardware and software components used to validate and test the proposed concepts, are released in open source and can be accessed from the official project repository (https://github.com/FPorreca/COCOWEARS (accessed on 27 October 2025)).

#### 2.1.2. Design of Reusable and Customizable Components

To achieve component reusability, each module within the COCOWEARS Framework must be structured following specific design rules. These standardized guidelines ensure that components, whether hardware or software, can be seamlessly integrated, swapped, or extended without affecting overall system functionality.

By adhering to these rules, developers gain the ability to achieve the following:

- Interchange hardware and software implementations of the same task, enabling flexibility in platform selection. For example, a KNN classifier or an IIR filter can be implemented either in software (CPU) or hardware (FPGA) depending on performance requirements.
- Expand functionality by designing new components or modifying existing ones while ensuring compatibility. This structured approach allows wearable systems to evolve without requiring extensive rework.
- Guarantee interoperability among all components, maintaining smooth communication across sensor nodes, processing units, and hardware accelerators regardless of their implementation type.

**Figure 1:**
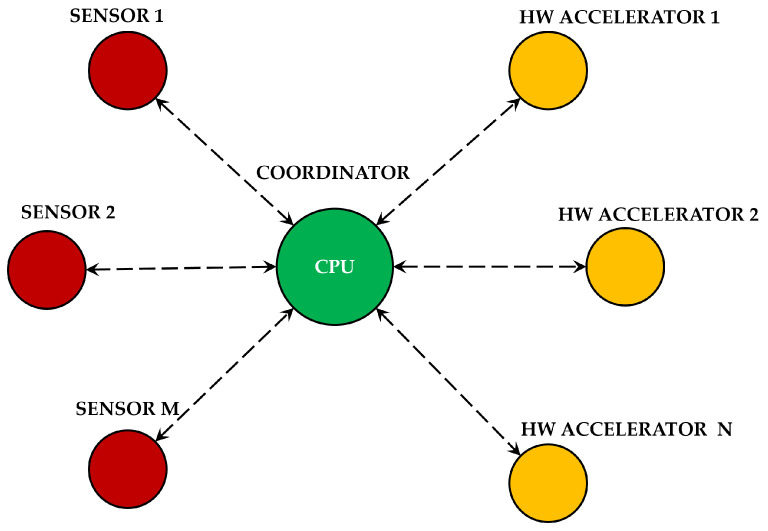
COCOWEARS architecture.

Similarly to what has been developed in [3], the architecture of the COCOWEARS framework follows a star topology (see Figure 1), with three main components: the coordinator, the sensor nodes, and the hardware accelerator nodes.

The coordinator, represented by the CPU of the SoC, plays a central role in the system’s operation. It manages the network and collects data from the sensor nodes using serial protocols. When data processing requires high computational power or low latency, the coordinator offloads computations to a hardware accelerator node implemented in the FPGA. Otherwise, it directly processes the data using software components.

**Figure 2:**
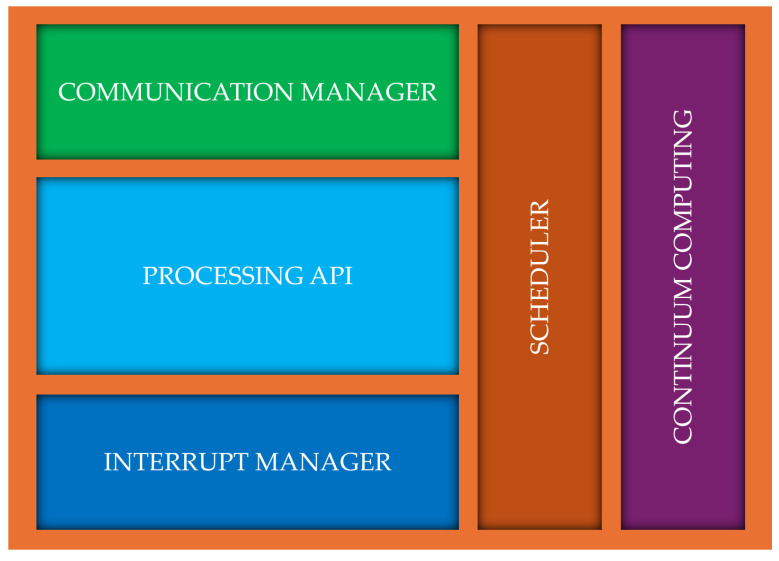
COCOWEARS coordinator functional architecture.

**Figure 3:**
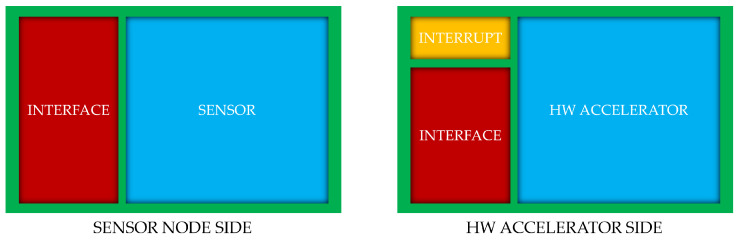
COCOWEARS node functional architecture.

Since the coordinator executes software components, it ensures proper integration between hardware and software, adapting execution based on the framework’s selection. If hardware accelerators are required, the coordinator is also responsible for data exchange between the CPU and the FPGA, optimizing the communication flow. The coordinator handles external communication, acting as a gateway in scenarios where cloud processing is essential or when integrating into a computing continuum architecture. This ensures that wearable systems remain scalable and interconnected, maintaining flexibility across local and distributed processing environments. The functional architecture of the COCOWEARS node is shown in more detail in Figure 2, and the functional architecture of COCOWEARS coordinator is shown in Figure 3.

By structuring the wearable system according to the components of the framework, the development process becomes significantly easier and the system much more scalable. This approach enables the creation of a versatile library containing hardware and software components, which can be utilized as needed based on specific requirements. To ensure seamless interchangeability, both hardware accelerators and software modules must expose common APIs. For instance, in the case of classifiers like kNN, the API is as follows:

int classify (Classifier c, int∗ instanceVector, int iSize,

int∗ trainingSet, int tSetSize);

Its use would look like the following instruction, where the declaration of the parameters passed to the function is not reported for the sake of brevity:

int class = classify (KNN, instanceV, INST_SIZE,

trainingS, TRAINING_SIZE);

Specifically:

- *Classifier*: Contains attributes related to the classifier to use, including the type (e.g., kNN, decision tree, …) and relevant parameters (e.g., number of nearest neighbors, in the case of kNN);
- *instanceVector*: A pointer to the input vector containing the new instance to be classified.
- *trainingSet*: A pointer to the training data needed to train the classifier model.

The input data and the returned result (i.e., the predicted class label) are consistent across both hardware and software implementations, ensuring uniformity. However, the source and handling of these data differ:

- *Hardware Implementation*: Data are sent to the hardware accelerator via the ARM Advanced eXtensible Interface (AXI)-Lite protocol, and the result is extracted from the hardware accelerator’s register through the same protocol.
- *Software Implementation*: The software routine processes the data locally and returns the result directly.

This consistent interface allows for the seamless replacement of hardware modules with software implementations and vice versa, without disrupting the overall system flow. The components of this library can be used by developers to build the system, allowing them to transparently utilize software functions or hardware accelerators. This means that developers can effectively employ hardware accelerators even without expertise in VHDL development.

#### 2.1.3. The Coordinator

The coordinator is the core of the system and is always present. It runs a C++ application specifically optimized to ensure high performance and reliability, orchestrating both software execution and hardware acceleration management. This application is designed as a modular system, integrating various software components that handle distinct functionalities:

- *Communication Manager*—A software routine responsible for handling wired and wireless network protocols, ensuring fast and secure communication with external sensors and hardware accelerators. The communication manager not only acquires the data from the sensors but is also in charge of configuring the sensors with the appropriate register settings when necessary.
- *Processing API*—A collection of software routines essential for the application, including operations like kNN classification, IIR filtering, and other processing tasks. These routines handle operations that can be executed more slowly, avoiding the need to send data to hardware accelerators and preventing the CPU from being bogged down, which could slow down other operations.
- *Interrupt Manager*—Manages interrupt signals originating from processing APIs (software routines) or hardware accelerators, ensuring timely response and efficient execution.
- *Scheduler*—Organizes the execution of various components based on the recommendations generated by the framework, ensuring efficient scheduling that prevents software routines from becoming a bottleneck for hardware accelerators and optimizes overall system performance. At the current stage, the proposed framework still allows software tasks to be scheduled using a simple interrupt-based mechanism, without assigning any specific priority. More complex and efficient scheduling mechanisms are planned to be developed and integrated in future work.
- *Continuum Computing Module*—Acts as the gateway for external communications, managing connections to cloud computing resources or enabling distributed processing within a computing continuum architecture when necessary.

By integrating these components, the coordinator efficiently balances hardware acceleration and software execution, ensuring seamless interoperability between modules while optimizing latency, processing speed, and resource allocation.

**Figure 4:**
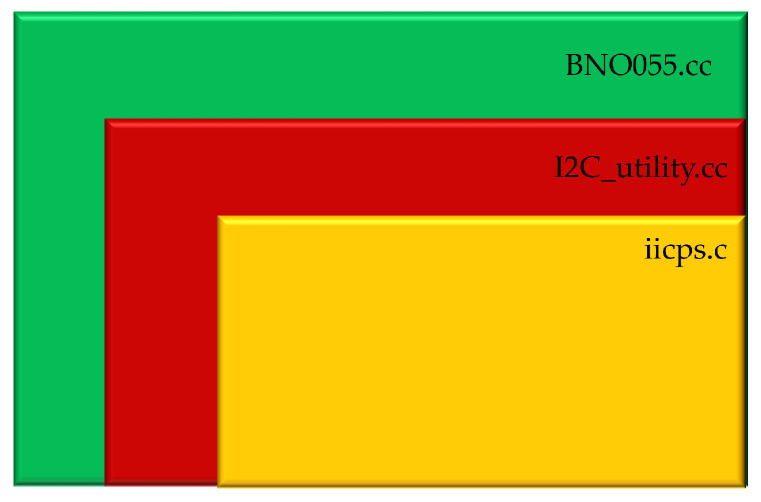
Level division of the software for communication.

To enhance software reusability, for the communication manager, we have developed a library for configuring and acquiring data from sensors that use $I^{2}C$ serial protocol. The library is organized in three level (see Figure 4): two levels are entirely reusable for any $I^{2}C$ sensor, and one level is sensor-specific. This approach reduces the designer’s customization effort when only the latter layer needs to be modified to adapt the system to the specific characteristics of a sensor featuring an $I^{2}C$ interface. For instance, this may involve adjusting the number of bytes to be transferred, which depends on the nature of the physical quantity measured by the sensor. It is worth noting that the $I^{2}C$ interface employs a standardized communication protocol, which is also commonly used in sensors implemented on flexible substrates—particularly designed for wearable systems [15]. Note that Figure 4 illustrates only the modular design of the $I^{2}C$ interface in the current prototype—chosen because the BNO055 sensor uses $I^{2}C$. However, the communication manager is a conceptual container, not a monolithic block: it hosts protocol-specific modules to support other standards (e.g., UART or SPI). The same layered architecture—separating the high-level, protocol-agnostic logic from low-level drivers—applies universally. Thus, replacing or adding support for, say, an SPI sensor (e.g., MAX6675) or a UART sensor (e.g., URM06) requires modifying only the low-level implementation, while preserving the common interfaces and keeping the rest of the system unchanged.

The first level consists of the library provided by Xilinx IICPS, which contains all the APIs needed to utilize the built-in IIC controllers of the off-the-shelf heterogeneous SoC families suitable to implement the proposed framework, such as the Zynq-7000 or Zynq-UltraScale, that interact directly with the hardware.

The second layer is a specially designed library that can be reused for any project utilizing the $I^{2}C$ protocol. By leveraging the functions in the IICPS library, it incorporates several helpful methods that shield the developer from certain technical details.

In particular, the main methods present in this library are the following:

- Begin (void): Takes care of initializing the processor’s $I^{2}C$ controller and configuring it according to the specific requirements, based on the parameters defined by the constructor method ($I^{2}C$ address, communication clock frequency).
- Read (u8 ∗MsgPtr, s32 ByteCount): Performs a read for a number of bytes specified by the *ByteCount* parameter. To perform the read, the signals carried by the SDA and SCL lines must be modified; this is done by the lower level iicps library.
- Write (u8 ∗MsgPtr, s32 ByteCount): Performs a write for a number of bytes specified by the *ByteCount* parameter. To perform the write, the signals carried by the SDA and SCL lines must be modified; this is done by the lower level iicps library.
- Write_then_read (u8 ∗write_buffer, size_t write_len, u8 ∗read_buffer, size_t read_len): Performs two successive operation without interrupting communication: write and then read. This method is the most useful and widely used because it is employed by the upper-level library whenever a sensor value needs to be obtained. When one wants to read the value of a specific register of an $I^{2}C$ slave device, the address of the register to be read is first sent, and then the contents of that register are read. The code for the method that performs what is described is reported in Appendix A.

The third and final level is tailored to the specific device you wish to communicate via $I^{2}C$ protocol, making it non-reusable. In this case, when developing a library for the BNO055 sensor, we list all the register addresses of this sensor in the header file. The methods in this library utilize this information, along with the methods provided by the lower-level library, to initialize, configure, and acquire data from the sensor with just a few lines of code. This approach has been widely adopted in the development of microcontrollers, where its effectiveness in ensuring flexibility and modularity is well-established. However, to the best of our knowledge, it has not yet been implemented on more complex platforms like those utilized in this work. This makes our effort a pioneering demonstration of the framework’s scalability and applicability to sophisticated systems, such as wearable devices, highlighting its potential to bridge the gap between compact embedded systems and intricate, high-performance platforms.

#### 2.1.4. The Sensor Node

Sensor nodes are critical components in any sensor network, serving as the primary source of data collection. These nodes can vary widely in their design and functionality, depending on the specific application and the type of data they are meant to collect. Each sensor node consists of two main components: the interface and the sensor itself.

The *interface* is responsible for communication with the rest of the system and supports various protocols such as $I^{2}C$, SPI, UART, as well as wireless standards like IEEE 802.15.4 [16] and Bluetooth. The selection of the appropriate protocol is based on factors like data rate, power consumption, and communication range. The *sensor* interacts directly with the physical environment, measuring parameters such as temperature, humidity, pressure, light, and motion. Analog sensors require an ADC (Analog-to-Digital Converter) to convert signals into a digital format, while digital sensors provide direct digital output to the system.

Before data collection can begin, each sensor requires configuration, which involves adjusting specific register values to set parameters such as measurement range, sampling rate, and power mode. The communication manager, operating on the coordinator side, is responsible for transmitting these configuration commands to the sensor nodes. COCOWEARS is structured to manage communication entirely in software through the CPU. Once the sensor data reaches the CPU, it can either be processed directly or sent to hardware accelerators for more efficient computation. This interaction follows a standardized ARM Advanced eXtensible Interface (AXI)-Lite protocol, ensuring reusability across different components. Specifically:

- The CPU transfers data to dedicated hardware registers within the FPGA via AXI Lite.
- The accelerators read the data from these registers and execute processing tasks.
- Once computations are completed, results are stored in new output registers, which the CPU can access through AXI Lite to retrieve the processed data.

By employing this structured approach, COCOWEARS ensures a seamless integration between software-driven communication and hardware-accelerated processing, enabling efficient execution of tasks while maintaining flexibility and scalability.

#### 2.1.5. The Hardware Accelerator Node

The hardware accelerators are developed in hardware description language (HDL) and implemented on the FPGA chip (see next paragraph for more details). They are responsible for processing the data from the sensors when the CPU lacks the computational power or when the task requires strict time constraints. Additionally, by offloading complex tasks from the CPU to the hardware accelerator, the overall power consumption of the system is reduced. This is particularly important in wearable applications, where battery life is a critical factor. Usually an hardware accelerator is composed of three main components:

- *Processing unit*: These component performs operations such as mathematical calculations, data encryption, signal processing, and more.
- *Memory*: Hardware accelerators often include memory that allow them to store processed data or used during calculation chain for partial results.
- *Communication Interfaces*: To interact with the rest of the system, hardware accelerators include communication interfaces. These interfaces can use various protocols, such as the Advanced eXtensible Interface (AXI) [17], to ensure seamless data transfer between the accelerator and other components of the SoC.

Usually, these components are regulated by an FSM (Finite State Machine) that manages all the control signals, ensuring that the hardware accelerator operates efficiently and effectively.

Notably, our approach is compatible with the presence of multiple wearable devices. For instance, in use case study presented in the following Section, both a localization system and a human activity recognition system were implemented on the same platform. We would like to emphasize that the HW/SW co-design approach allows the implementation, within the programmable logic section, of hardware architectures that can operate in parallel and concurrently, each performing its own data processing independently, without the need to schedule different processes on the same hardware at separate time instants. The resulting outputs can then be jointly processed within an information fusion paradigm to provide a more structured and comprehensive understanding.

### 2.2. The Use Case

**Figure 5:**
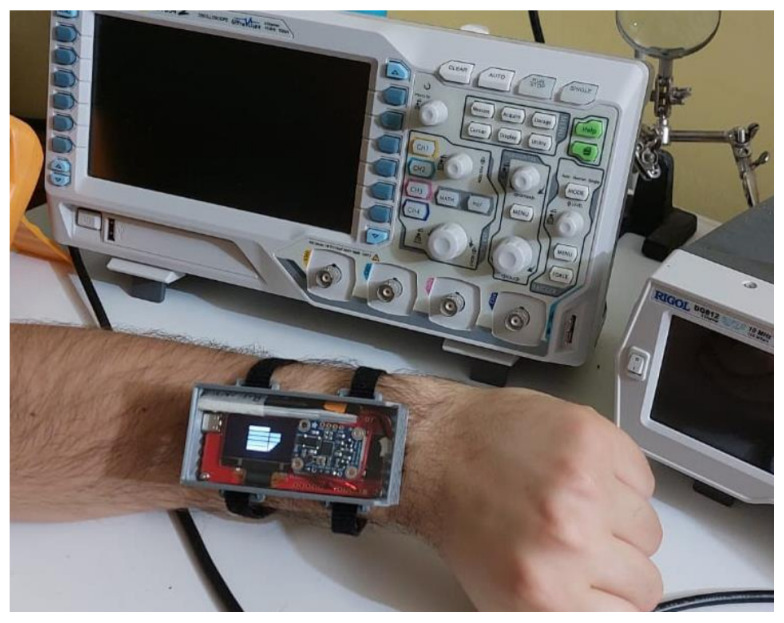
Wrist-worn wearable prototype system in its 3D-printed housing.

Serving as the bridge between COCOWEARS’ conceptual architecture and its real-world validation, we selected the Digilent Cora Z7-07S development board as our reference platform. This low-cost, off-the-shelf solution—built around the Xilinx Zynq-7000 APSoC—directly reflects the framework’s abstractions. It integrates a general-purpose processor—or Processing System (PS)—and a Programmable Logic (PL) fabric within a single chip: the on-chip ARM Cortex-A9 core plays the role of the coordinator, running the C++ orchestrator and handling all software routines, whereas the programmable FPGA fabric hosts the hardware accelerators. At its heart lies the XC7Z007S-1CLG400 SoC, combining a single-core 667 MHz Cortex-A9 processor with reconfigurable logic for true HW/SW co-design [18]. Thanks to its extensive GPIO array and dedicated $I^{2}C$, SPI, and UART connectors, the Cora Z7-07S lets us plug in a variety of analog and digital sensors perfect for proving the reusability of COCOWEARS in a wearable context. Expansion headers support off-the-shelf Wi-Fi and Bluetooth Low Energy (BLE) radios, closing the loop on COCOWEARS’ computing continuum vision by enabling seamless cloud offloading when on-device resources are stretched. The chosen use case concerns a system for real-time location monitoring and activity recognition of people within an indoor environment. Localization was performed using ultra-wideband (UWB) technology and trilateration algorithms, while activity recognition was based on data from a magneto-inertial sensor processed using a kNN-type machine learning algorithm. A wearable prototype was developed to implement this system, designed to be worn like a smartwatch on the dominant wrist. To minimize intrusiveness, a compact 3D-printed casing was created for housing the device. The prototype includes a BNO055 smart sensor, which integrates an accelerometer, gyroscope, magnetometer, and an embedded microcontroller running sensor fusion software. For localization, ESP32 MaUWB boards equipped with UWB transceivers and wireless connectivity (Wi-Fi/Bluetooth) were used to collect and transmit positional data by communicating with fixed anchors in the environment. Sensors are housed within the case and connected externally to the development board via a flexible cable. The case, sealed by its polycarbonate cover, is shown in Figure 5 through which one can see the sensors and the display showing the university logo.

**Figure 6:**
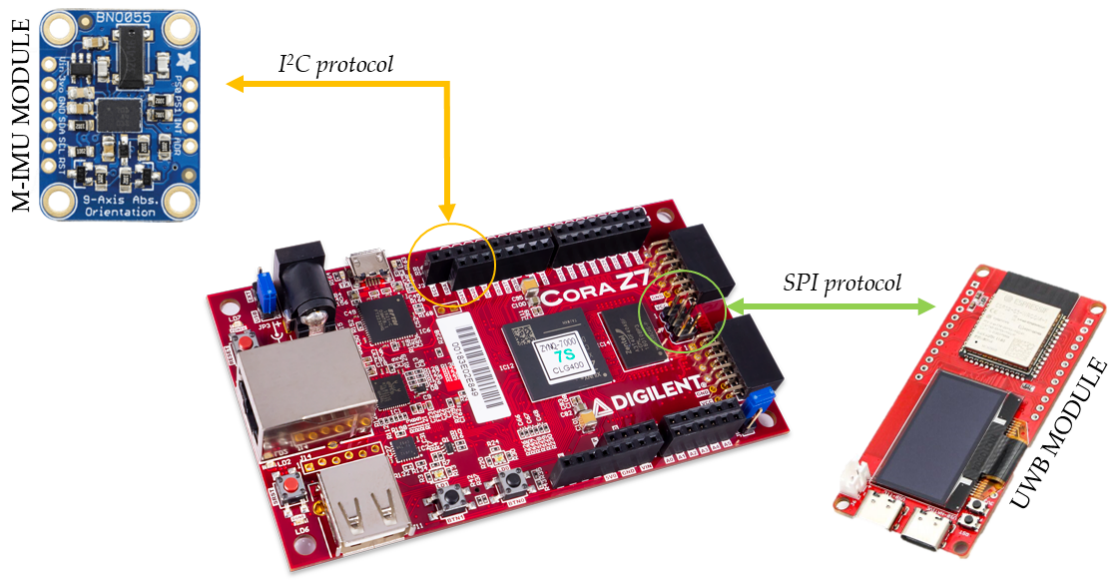
Sensors connection diagram.

The ESP32 MauUWB board for localization and the magneto-inertial sensor BNO055 constitute the sensor node, according to the COCOWEARS architecture shown in Figure 1. The BNO055 sensor integrate an hardware $I^{2}C$ communication interface, so the communication manager of the coordinator can interact directly with the sensor. The UWB chip for localization is connected to a ESP32 microcontroller, so the communication manager of the coordinator interact first with with the ESP32 microcontroller and then, the ESP32 microcontroller manage the UWB chip. The communication between the coordinator connection manager (master) and the microcontroller (slave) is executed via SPI protocol. In this case, the ESP32 integrates the interface that was made using the Espressif software API SPI Slave Driver. The wiring connection between the sensors and the development board are made according to the schematic shown in Figure 6. This setup utilizes the dedicated physical pins for the serial communication protocol of the Cora Z7-07S. By following the schematic, each sensor is correctly interfaced with the development board, ensuring reliable data transmission and optimal performance of the system.

The sensor node connected with the board is managed by the coordinator, i.e., the Arm core of the SoC. The Communication Manager component of the coordinator directly interacts with the hardware of the main development board and exchanges data with the sensors using serial protocols. Specifically, for the chosen use case, the communication manager handles the communication between the magneto-inertial senor with $I^{2}C$ interface and the localization sensor with the SPI protocol.

Once data are acquired, they must be processed. A priori, it is not possible to determine whether it is more convenient to process the data by hardware acceleration on FPGA or by a software routine on CPU. Our approach is to develop both solutions and adopt the one most convenient in terms of resources consumption (battery power) and performance (speed of execution). The metrics that characterize a solution were extracted through software simulations. Interchangeability between a software and hardware solution is ensured through the interface, which must be the same for both solutions.

In this case, the operation of acquiring data from the sensors and sending them to the hardware accelerator is managed sequentially, eliminating the need for the scheduler and the interrupt manager. Additionally, the gateway, which typically plays a role when local hardware or software resources are insufficient for processing tasks, is not used in this setup. This is because the selected development board is capable of executing all operations locally, whether entirely in software, entirely in hardware, or through a hybrid approach.

### 2.3. HW Accelerator Design

For the chosen use case, two hardware accelerators have been developed, one for the trilateration calculus and one for the kNN classification. These accelerators were designed using VHDL language and rigorously simulated and tested in the Xilinx Vivado development environment to verify their performance and reliability. Following successful validation, they were implemented on the physical prototype. In this implementation, the operation of acquiring data from the sensors and sending it to the hardware accelerators was managed sequentially. Consequently, components such as the interrupt controller were not utilized and implemented, simplifying the system’s design for this specific application.

#### 2.3.1. Trilateration

Once the CPU acquires the data from the sensors, it transfers the information to the hardware accelerator using the AXI protocol. For the chosen use case, at least three anchors are needed to determine the position of the tag in space. Each anchor is characterized by three parameters: a pair of coordinates representing its spatial position and a distance measurement from the tag. These values are processed by the hardware accelerator according to the equations presented earlier, ultimately yielding the x and y coordinates of the tag.

The accelerator was designed using fixed-point arithmetic, considering the characteristics of the system generating the data to be processed. This approach helps reduce resource consumption and optimize both execution speed and power consumption. Specifically, the accuracy of the localization system, which is approximately 25 cm in this case, was taken into account. Consequently, an appropriate word length was chosen to ensure that computational accuracy is maintained throughout the processing chain.

For data processing, cascaded DSPs were utilized to take advantage of the dedicated fast hardware interconnections available for these specialized devices, thereby enhancing the computational speed. These DSP blocks, specifically the DSP48E1, are advanced digital signal processing units that feature integrated functionalities such as multipliers, adders, and accumulators. Their compact structure and optimized design make them ideal for accelerating computational tasks in FPGA implementations. Additionally, Xilinx IP Core dividers with a radix-2 algorithm were employed. The minimum clock period of the circuit is 5 ns, corresponding to a maximum operating frequency of 200 MHz. The circuit has an initial latency of 50 clock cycles, after which it can generate a useful result every clock cycle, thanks to the perfectly balanced pipeline.

#### 2.3.2. kNN

Before implementing the kNN algorithm on hardware, the concept of using a single magneto-inertial sensor placed on the user’s dominant wrist was validated through simulations with Weka software v3.8 (https://ml.cms.waikato.ac.nz/weka (accessed on 27 October 2025)). From the simulation, the system can distinguish with about 94% accuracy between sitting and walking activities.

The decision to use the kNN algorithm for classification in this work was based on its widespread popularity in the literature as a technique for human activity recognition (HAR). Numerous studies have demonstrated its effectiveness in classifying movement patterns using wearable sensor data, making it a well-established choice for this type of application. While other machine learning algorithms could achieve similar objectives, kNN remains a frequently adopted method due to its simplicity, adaptability, and strong performance in recognizing human activities. Furthermore, the choice to rely on a single sensor was made to ensure that the device remains less intrusive for the user and to simplify its development within the context of the chosen use case. This approach balances functionality and practicality while maintaining alignment with the overarching goals of the system.

The hardware accelerator used for kNN classification processes input data transferred via the AXI Lite Protocol. It utilizes a total of 10 registers: three registers are assigned to each of the sensors (accelerometer, magnetometer, and gyroscope) for data acquisition, and one register is reserved for storing the classification results.

For the development of the computational module, low-power techniques such as clock gating and fixed-point arithmetic were employed. The word length was carefully chosen based on the characteristics of the dataset to ensure efficient processing. DSPs were again utilized to calculate the distance, leveraging the dedicated internal links to enhance execution speed. The dataset, comprising 7206 instances, was stored on-chip using the BRAM slices of the FPGA device.

**Figure 7:**
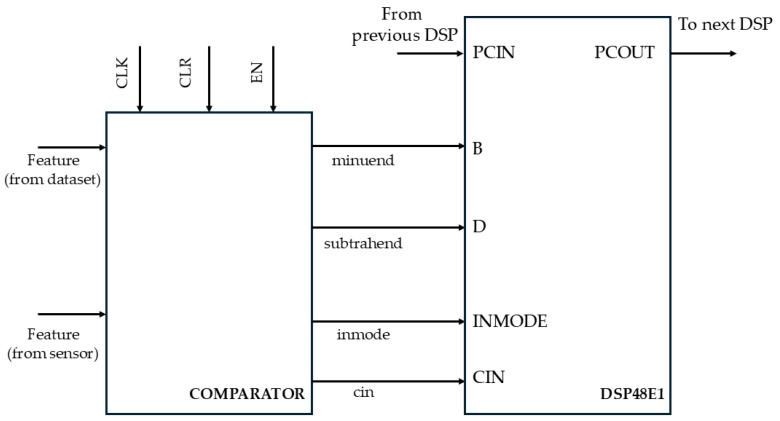
Manhattan distance hardware accelerator schematic.

The Weka simulations identified the Manhattan distance as the most suitable metric for this case. To implement it in hardware, a dedicated absolute value function was developed. The architecture leverages the DSP configuration capabilities to compute the absolute difference without disrupting the high-speed PCIN-PCOUT path. This is achieved by adjusting the INMODE and carry-in signals to perform the operation as a sum, avoiding the need for external logic or two’s complement computation. This solution improves speed and reduces area and power consumption. Figure 7 illustrates a DSP stage used for comparing input features. The developed circuit performs a classification in 7226 clock cycles. The minimum clock period is 9ns, which corresponds to a maximum operating frequency of approximately 111MHz. It is worth noting that the implemented hardware accelerator does not cause any loss of classification accuracy, despite being designed to operate with fixed-point arithmetic. This behavior is typical of machine learning applications, which are inherently robust to the noise introduced by reduced numerical precision in the underlying data representation.

### 2.4. Software Routines Design

Two modular APIs were developed for localization and kNN classification, implementing a software-only approach on the same CPU platform used in the hardware/software framework. This allows consistent and realistic comparisons in terms of power and performance. While designed to mimic a standalone microcontroller setup, the APIs are also reusable within the framework—enabling hybrid implementations where, for instance, trilateration runs in software and classification on hardware accelerators. The C++ modular structure ensures portability across platforms, maintaining integration with the overall system architecture.

## 3. Results

To highlight the advantages offered by the proposed framework, the previously described case study was implemented using two different approaches: one based on the mixed hardware–software design and the other employing a purely software-based solution. In Table 2, these approaches are identified by the labels Type A and Type B, respectively. Type A tests were carried out using a hardware solution that not only employs dedicated hardware accelerators but also features a CPU responsible for acquiring sensor data and forwarding it to the accelerators. In contrast, Type B tests were carried out using only software APIs, representing a traditional software-based development methodology without utilizing the framework. Moreover, for each implementation type, a design exploration has been carried out by setting the operating clock frequency to different values.

**Figure 8:**
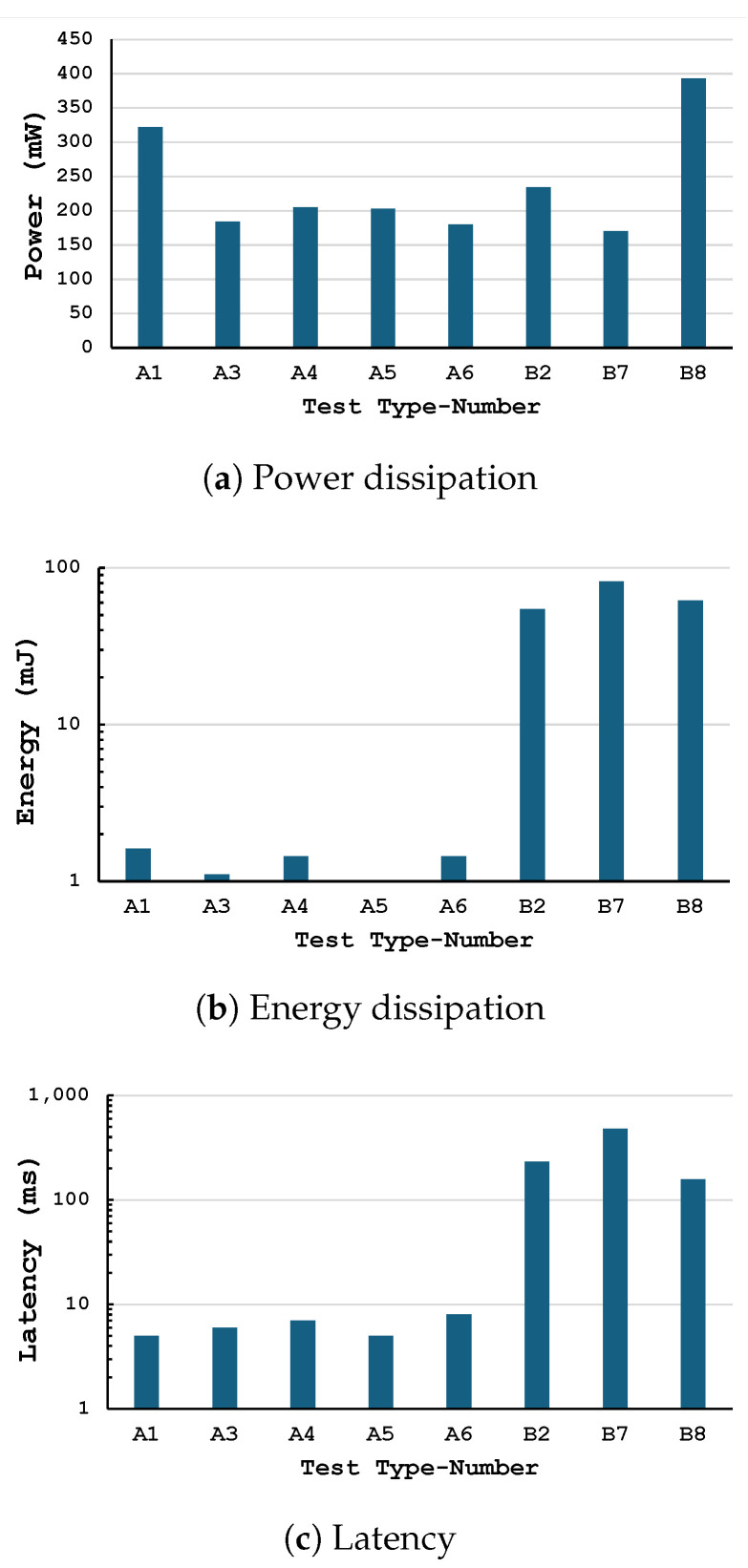
Performance of the designed WCS for each implementation scenario.

In this context, PS refers to the frequency of the Processing System (i.e., the CPU portion of the Zynq SoC), while PL refers to the frequency of the Programmable Logic, i.e., the hardware accelerator portion. Naturally, the software APIs run on the CPU in the PS domain, while the hardware accelerators execute in the PL domain at their configured clock rate. Power consumption was measured using the same board for both Type A and Type B tests to ensure comparability. All parasitic components on the development board (e.g., external memory, power regulators, interfacing circuitry) are present in both cases, introducing a common offset that is not measurable in isolation but affects both measurements equally. By keeping hardware and system conditions identical, we minimize external variation, isolating the power savings and performance differences due solely to the use of hardware acceleration versus software execution. Table 2 and Figure 8 summarize the information regarding latency and power dissipation for the different analyzed scenarios (Type A and Type B). The main result to highlight is that the solutions based on the COCOWEARS framework exhibit significantly lower latency compared to traditional solutions that rely solely on software routines. As an example, the execution time of the k-NN algorithm can be reduced by up to a factor of 96. Similarly, it can also be observed that a hardware–software co-design approach provides advantages in terms of power dissipation. In fact, when comparing two Type A and Type B solutions operating at the same PS clock frequency, it can be noted that the former exhibits lower power consumption, which, according to the results reported in Figure 8, can be reduced by up to 21%. This demonstrates the effectiveness of the proposed approach for the design of wearable systems capable of meeting stringent latency and power constraints, while being efficiently implementable within the edge computing paradigm.

For each scenario, several operating conditions were also investigated, in which the clock frequencies of the Processing System (PS) and the Programmable Logic (PL) assume different values. The purpose of this analysis is to highlight how the system metrics vary as a function of the configured clock frequency and how it is possible to achieve a trade-off between power dissipation and latency. It is readily visible that, as expected, increasing the clock frequency (either in the PS or in the PL) leads to a higher power consumption and a lower latency.

For example, consider tests number 1 and 3, both implemented according to the COCOWEARS framework. The clock frequency at which the hardware accelerators in the PL operate is set to the same value (i.e., 50 MHz), while the clock frequency of the PS is set to 650 MHz (the maximum value) and 240 MHz, respectively. As expected, the latency in Test No. 1 is lower—by approximately 38% and 17% for the trilateration and kNN algorithms, respectively. Conversely, the power consumption is higher by about 75%. Nevertheless, from the perspective of energy dissipation, the situation is not straightforward, since modifying the clock period does not affect the dynamic component of energy consumption but only the static one. Moreover, it obviously influences the total latency required by the task to complete all its processing operations. From the data reported in Table 2, it can be inferred that the solution dissipating the least amount of energy (i.e., 202.9 mW × 5.0075 ms = 1.016 mJ) is the one identified as Test No. 5, which corresponds to the implementation of the proposed framework with the PS and PL clock frequencies set to 240 MHz and 100 MHz, respectively. It is worth noting that the three scenarios where the proposed framework has not been adopted, namely, Tests No. 2-7-8, show an energy consumption considerably higher: it can be as high as 80× than that showed by the scenario Test No. 5.

In this case study, extreme environmental conditions (e.g., high/low temperature or humidity) do not significantly affect the obtained outputs, neither in the localization nor in the human activity classification. However, it worth noting that certain applications may require such information, in which case, once the sensor data are acquired by the wearable system, such corrections can be implemented within the software routines or the hardware architecture, depending on whether the module is implemented as software code or as a hardware accelerator.

## 4. Discussion

The adoption of the framework has significantly facilitated the development of this system and proves applicability for the creation of other systems as well. The software APIs and hardware accelerators developed for this project can be organized into a library, making them reusable for future projects where similar operations are required. Additionally, new components can be developed following the rules of the framework, enabling the extension of functionalities and further adaptability to different use cases. The system performances were measured in different scenarios, using either software APIs or hardware accelerators, and setting different clock frequencies for the CPU and the hardware computing modules. The system performances were measured in different scenarios, using either software APIs or hardware accelerators, and by varying the clock frequencies for both the CPU and the hardware computing modules to assess changes in power dissipation. In the first scenario, all computations were performed entirely on the CPU, using software APIs to simulate a traditional development approach. In contrast, the second scenario leveraged hardware accelerators, where the CPU acts only as a coordinator, delegating all computational tasks related to sensor data processing to specialized accelerators.

**Figure 9:**
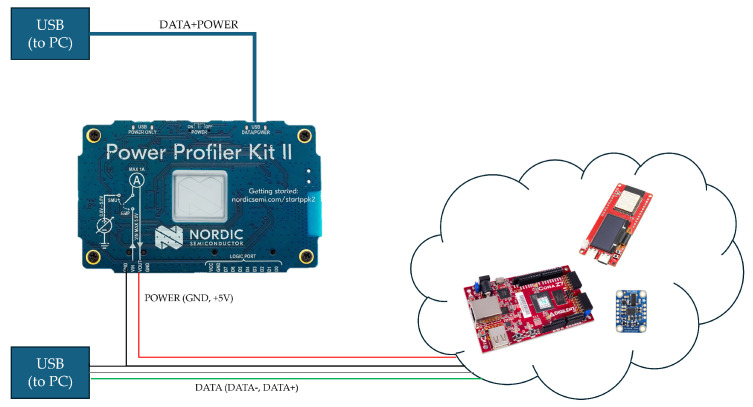
Power analysis setup.

The maximum sampling rate supported by the sensors is 100 Hz, which is more than enough to monitor human activity. As can be seen from the Table 2, Test No. 8 uses a CPU frequency of 650 MHz, and, despite being so high, the trilateration calculation plus kNN classification take c.a. 157 ms to be performed, i.e., the system performs the calculations at a maximum frequency of c.a. 6 Hz, which is not enough to take full advantage of the adopted sensors. That solution also draws about 393 mW calculated as the difference between the value absorbed by the device during code execution and the value absorbed during the resting state (measured with Nordic Semiconductor’s PPK II, see Figure 9). On the other hand, from test 6, it can be seen that the solution adopting a hardware-software co-design technique and the COCOWEARS architecture can achieve a computational latency of only 8ms i.e., with a potential frequency of 125 Hz. Moreover, this solution reduces the power consumption by up to about 50%.

## 5. Conclusions and Future Works

This work presents a hardware–software co-design methodology for wearable computing systems, centered on the COCOWEARS framework. Its aim is to streamline development while ensuring scalability, reusability, and energy efficiency. Unlike ad-hoc approaches often used in wearable design, this structured framework supports modular development across heterogeneous platforms, combining the flexibility of MCUs with the performance of FPGAs. The case study demonstrated the framework’s effectiveness, achieving significant speedups in real-time machine learning tasks and reducing power consumption by over 50%. Its architecture also enables easy integration of new processing modules, making it suitable for a wide range of future applications. The case study has been implemented on a heterogeneous System-on-Chip (SoC). The developed software and hardware components have been described in C and Very large scale integration (VLSI) Hardware Description Language (VHDL), respectively, in order to ensure high portability across different platforms that integrate a mixed MCU/FPGA architecture within the same chip. Overall, COCOWEARS offers a solid foundation for designing next-generation wearable systems, promoting low-power edge computing and accelerating time-to-deployment through standardized and scalable design practices. The framework, proposed as an open-source project, is in its fully active development. We are planning to include artificial intelligence mechanisms such as Convolutional Neural Networks (CNNs) by providing hardware library components (e.g., edge Tensor Processing Units such as Google Coral or Edge GPUs such as NVIDIA Jetson Nano) that implement well-known neural network models suitable for typical wearable applications and to characterize them in terms of energy consumption, computational power, and resource utilization. Future developments will also focus on the design and characterization of modules dedicated to data protection mechanisms. Beyond the integration of hardware and software components for data encryption, the proposed co-design approach is well suited to the implementation of other critical elements in this context, such as True Random Number Generators (TRNGs) [19] or Physical Unclonable Functions (PUFs) [20]. These components exploit unique hardware-specific features—such as jitter and metastability—as sources of entropy, or rely on unpredictable process variations to ensure that each implementation is uniquely identifiable. Finally, the fact that the proposed approach leverages the presence of hardware modules to implement certain computational steps provides the opportunity to incorporate run-time power reduction mechanisms during time windows in which data acquisition and processing are not required. This is made possible by integrating hardware-oriented power management techniques, such as data gating and clock gating, during sleep-mode phases. As future work we also plan to investigate the possibility of extending the framework to multi-user scenarios typical of collaborative wearable computing systems.

## Figures and Tables

**Table 1:** SoTA WCS vs. COCOWEARS WCS.

Aspect	State-of-the-Art (SoTA) WCS	COCOWEARS WCS
Performance	Limited by microcontroller capabilities	Enhanced with FPGA
Power Efficiency	Higher power consumption due to cloud offloading	Reduced with local processing
Development Complexity	Simplified with software-only tools	More challenging due to hardware-software codesign
Scalability	Limited for computationally heavy tasks	Highly scalable with support for new functionalities
Latency	High due to cloud dependency	Low with on-device execution

**Table 2:** Power analysis results. A: with HW accelerator, B: without HW accelerator.

Test No.	Test Type	PS [MHz]	PL [MHz]	Trilat. [ms]	kNN [ms]	Power [mW]
1	A	650	50	0.005	5	321.9
2	B	240	ND	0.05	232	234.45
3	A	240	50	0.008	6	184.2
4	A	240	1	0.008	7	205.35
5	A	240	100	0.0075	5	202.9
6	A	80	50	0.014	8	180.2
7	B	80	ND	0.109	480	170.4
8	B	650	ND	0.032	157	393.15

## Data Availability

All data and source code used in this study are openly available at the following GitHub repository: https://github.com/FPorreca/COCOWEARS, accessed on 27 October 2025.

## Appendix A

The Write_then_read method in the I2C_utility.cc library:

int I2CDevice::write_then_read(u8 ∗write_buffer, size_t write_len,

                 u8 ∗read_buffer, size_t read_len) {

     xil_printf(‘‘Running write_then_read Operation\r\n’’);

     if(write(write_buffer, write_len) != XST_SUCCESS) {

          xil_printf(’’write_then_read Failed\r\n’’);

     return XST_FAILURE;

}

return read(read_buffer, read_len);}
